# Effects of an Artificial Intelligence–Assisted Health Program on Workers With Neck/Shoulder Pain/Stiffness and Low Back Pain: Randomized Controlled Trial

**DOI:** 10.2196/27535

**Published:** 2021-09-24

**Authors:** Tomomi Anan, Shigeyuki Kajiki, Hiroyuki Oka, Tomoko Fujii, Kayo Kawamata, Koji Mori, Ko Matsudaira

**Affiliations:** 1 Department of Occupational Health Practice and Management Institute of Industrial Ecological Sciences University of Occupational and Environmental Health, Japan Kitakyushu Japan; 2 Cenxus Occupational Physicians' Firm Tokyo Japan; 3 Advanced Occupational Health Research and Consulting Inc Tokyo Japan; 4 Department of Medical Research and Management for Musculoskeletal Pain 22nd Century Medical and Research Center, Faculty of Medicine The University of Tokyo Tokyo Japan

**Keywords:** neck pain, shoulder pain, shoulder stiffness, low back pain, musculoskeletal symptoms, digital intervention, mobile app, mHealth, eHealth, digital health, mobile phone

## Abstract

**Background:**

Musculoskeletal symptoms such as neck and shoulder pain/stiffness and low back pain are common health problems in the working population. They are the leading causes of presenteeism (employees being physically present at work but unable to be fully engaged). Recently, digital interventions have begun to be used to manage health but their effectiveness has not yet been fully verified, and adherence to such programs is always a problem.

**Objective:**

This study aimed to evaluate the improvements in musculoskeletal symptoms in workers with neck/shoulder stiffness/pain and low back pain after the use of an exercise-based artificial intelligence (AI)–assisted interactive health promotion system that operates through a mobile messaging app (the AI-assisted health program). We expected that this program would support participants’ adherence to exercises.

**Methods:**

We conducted a two-armed, randomized, controlled, and unblinded trial in workers with either neck/shoulder stiffness/pain or low back pain or both. We recruited participants with these symptoms through email notifications. The intervention group received the AI-assisted health program, in which the chatbot sent messages to users with the exercise instructions at a fixed time every day through the smartphone’s chatting app (LINE) for 12 weeks. The program was fully automated. The control group continued with their usual care routines. We assessed the subjective severity of the neck and shoulder pain/stiffness and low back pain of the participants by using a scoring scale of 1 to 5 for both the intervention group and the control group at baseline and after 12 weeks of intervention by using a web-based form. We used a logistic regression model to calculate the odds ratios (ORs) of the intervention group to achieve to reduce pain scores with those of the control group, and the ORs of the subjective assessment of the improvement of the symptoms compared to the intervention and control groups, which were performed using Stata software (version 16, StataCorp LLC).

**Results:**

We analyzed 48 participants in the intervention group and 46 participants in the control group. The adherence rate was 92% (44/48) during the intervention. The participants in the intervention group showed significant improvements in the severity of the neck/shoulder pain/stiffness and low back pain compared to those in the control group (OR 6.36, 95% CI 2.57-15.73; *P*<.001). Based on the subjective assessment of the improvement of the pain/stiffness at 12 weeks, 36 (75%) out of 48 participants in the intervention group and 3 (7%) out of 46 participants in the control group showed improvements (improved, slightly improved) (OR 43.00, 95% CI 11.25-164.28; *P*<.001).

**Conclusions:**

This study shows that the short exercises provided by the AI-assisted health program improved both neck/shoulder pain/stiffness and low back pain in 12 weeks. Further studies are needed to identify the elements contributing to the successful outcome of the AI-assisted health program.

**Trial Registration:**

University hospital Medical Information Network-Clinical Trials Registry (UMIN-CTR) 000033894; https://upload.umin.ac.jp/cgi-open-bin/ctr_e/ctr_view.cgi?recptno=R000038307.

## Introduction

Musculoskeletal symptoms of the neck and shoulder and low back pain are common health problems in the working population [1,2]. In Japan, shoulder stiffness and low back pain are the most common somatic symptoms. In men, low back pain is the most common, followed by stiff shoulders; in women, the order is reversed—stiff shoulders rank first, while low back pain ranks second [3]. Health problems in employees incur substantial costs in terms of medical care expenditure and poor work productivity, which is marked by absenteeism and presenteeism [4,5]. Absenteeism is defined as health-related absence from work, and presenteeism is the condition where employees are physically present at work but are unable to fully engage themselves [4,5]. A recent survey reported that neck and shoulder stiffness followed by low back pain were the leading causes of presenteeism in Japanese workers [5].

The causes of most musculoskeletal symptoms are not clearly known. However, primary neck/shoulder pain/stiffness and nonspecific low back pain are related to poor posture and psychological stress. Neck/shoulder stiffness can be classified into primary or secondary complaints. The causes of secondary neck/shoulder stiffness or pain include cervical spine diseases, glenohumeral joint diseases, cardiovascular diseases, eye fatigue, and temporomandibular disorder [6]. The causes of primary neck/shoulder stiffness or pain are not well known. However, hemodynamics in the trapezius muscle is expected to be involved [7,8]. Besides, head-down posture, psychological factors [9], and physical inactivity [10] are related to chronic neck and shoulder pain.

Low back pain is mainly classified into 2 categories: specific and nonspecific. Specific low back pain occurs when the symptoms are caused by a specific pathophysiological mechanism such as lumbar disk herniation, infection, osteoporosis, rheumatoid arthritis, fracture, or tumor [11]. It accounts for only about 10% of all low back pain cases [11]. In contrast, about 90% of patients with low back pain experience nonspecific low back pain, where the symptoms do not have a clear and specific cause [11].

Several studies have reported methods to minimize the discomfort from such symptoms—a combination of exercises and psychological approaches seems effective for patients with musculoskeletal symptoms. Moderate-to-strong evidence suggests that exercise therapy is effective in relieving pain and improving function in musculoskeletal disease [12-18]. However, there seems to be little evidence on the types of exercises and programs that are effective in relieving musculoskeletal pain [12,14,15,19]. There are also several psychological treatments or therapies for musculoskeletal symptoms [12]. In a study on patients with chronic low back pain, both groups (one that received only exercise therapy and another that received a combination of cognitive behavioral therapy and exercise therapy) showed improvements in pain intensity and quality of life compared to baseline [20]. Patients with chronic pain who received acceptance and commitment therapy, a third-generation cognitive behavioral therapy based on mindfulness, experienced minor improvements in pain intensity and the degree of depressive symptoms and dysfunction and moderate improvements in the degree of anxiety and disability caused by pain compared to patients in the normal treatment group and control group [21].

One problem with exercise therapy is the low level of adherence to the prescribed exercises. Two systematic reviews reported that up to 70% of participants did not adhere to the prescribed exercises [22,23]. The lower the adherence to exercise, the lesser effective is the therapy [24]. Therefore, adherence is a critical factor that determines the outcomes of the intervention process [25]. The type of exercise does not seem to influence adherence [25], but support from experts seems important [26,27]. Factors that enhance the adherence to exercise programs include the attractiveness of the programs, feedback from and interaction with experts, evaluation of patients’ performance, and the feeling of being supported by experts [24]. Supervised exercises, review sessions, and visual or audio aids are also effective [25]. Additional support such as phone calls, email reminders, and text messages promotes the engagement of digital interventions [28-33]. In Japan, the medical system has not been able to provide sufficient services for such conditions. Some enthusiastic medical professionals, preventive medical services, and occupational health services provide care for people with functional impairments such as musculoskeletal symptoms. In most other situations, patients need to look for ways to improve their symptoms on their own.

Currently, digital health programs using smartphones, tablets, and computers are relatively inexpensive and are widely accepted, especially by the young and the middle-aged. Three systematic reviews involving musculoskeletal symptoms that included a plethora of studies with digital interventions, for example, mobile phone apps, websites, and web-based software have been performed [28,34,35]. About half of these showed that digital interventions were effective in reducing low back pain [28,30-32,36,37]. There are limited studies on digital interventions for stiff neck/shoulders; an intervention for shoulder stiffness using a software program that promoted regular breaks did not show a significant effect on the severity and frequency of the symptoms [38]. Besides, 65% of smartphone apps that perform self-monitoring and self-managing of chronic pain have been developed without the involvement of experts and proper supporting evidence [39]. Conversely, it has also been suggested that digital interventions may have possibilities to improve adherence in the target population [26]. Some studies including digital health interventions have shown enhanced self-management and adherence to medications in patients with asthma, chronic obstructive pulmonary disease, hypertension, and diabetes [40-43].

The aim of this study was to improve neck/shoulder pain/stiffness and low back pain of workers who experienced those symptoms by continuing to do exercises that included stretching and mindfulness. As a measure of encouraging them to continue the exercises, we provided them with secaide Ver.0.9 [44], an artificial intelligence (AI)–assisted interactive health promotion system using a mobile messaging app (the AI-assisted health program). We hypothesized that this digital intervention would support participants to continue exercising and enhance their adherence to the exercises, resulting in greater improvement of symptoms. To the best our knowledge, this is the first study to use a chatbot as a health care support measure through a messaging app to improve the musculoskeletal symptoms of workers.

We implemented a randomized controlled trial (RCT) among workers with either neck/shoulder pain/stiffness or low back pain or both in a company setting to evaluate the improvement of musculoskeletal symptoms by using an AI-assisted interactive health promotion system through a mobile messaging app to habituate exercise.

## Methods

### Study Design

This study was a two-armed, randomized, controlled, and unblinded trial on workers with either neck/shoulder pain/stiffness or low back pain or both. We set the intervention and control groups, and the participants of the intervention group used the AI-assisted health program for 12 weeks and those of the control group continued their regular exercise routine at their workplace. We assessed the subjective severity of the neck and shoulder pain/stiffness and low back pain in both the intervention and control groups at baseline and 12 weeks immediately after the intervention. We provided an explanatory document regarding the study to the applicants and obtained informed consent. This trial was conducted with the approval of the ethics committee of the University of Tokyo Hospital (ID 12035) and the ethical review of the target company. This trial was registered at University hospital Medical Information Network-Clinical Trials Registry (ID 000033894). This RCT was performed based on CONSORT-EHEALTH (Consolidated Standards of Reporting Trials-eHealth) guidelines.

### Study Population

We conducted this study in a company that develops, designs, and manufactures precision electronic components and that has approximately 2200 employees. Some employees were manufacturing engineers who managed and supervised the manufacturing process of the precision electronic components, but most employees were white-collar employees who were engaged in the design, development, or clerical work of the product. The occupational health staff of the company recruited employees with remarkable musculoskeletal symptoms, either or both neck/shoulder stiffness/pain and low back pain. They chose participants based on data from the periodic health survey conducted by the target company. The company performs the survey once a year to check the physical and mental health conditions of the employees. The employees used a specific URL to sign into the health check system and answer specific questions, according to the instructions provided. We targeted those who answered “frequently” or “almost always” in either of the following questions in the survey:

1. How often do you experience neck/shoulder stiffness?

(1) Almost never, (2) Occasionally, (3) Frequently, (4) Almost always

2. How often do you have low back pain?

(1) Almost never, (2) Occasionally, (3) Frequently, (4) Almost always

They recruited applicants by email notifications between September 3 and 14, 2018. The inclusion criteria were as follows: employees aged 20-64 years, who had their own cell phones and the apps could be installed on the phones, and who understood the purpose and agreed to the publishing of the contents and results of the study. The exclusion criteria were as follows: employees who disagreed with the study, were pregnant or may have been pregnant, had cardiopulmonary diseases, participated in other clinical trials, and had any other obvious disabilities or exercise restrictions.

### Randomization

A total of 121 employees applied for the study. After we confirmed that the applicants met all the inclusion criteria and did not violate any of the exclusion criteria, all 121 participants were randomized to 2 groups by generating random numbers on a computer and stratified by 10-year age groups and separated by the age group with a 1:1 allocation ratio.

### Intervention

We held an initial training session for the applicants to explain the purpose of the study and obtained informed consent. We also explained to the participants of the intervention group how to install and use the AI-assisted health program on their cell phones to inform them a Quick Response code and a passcode to access the program on September 26 and 27, 2018. After that, they started to use the program. The control group continued with their regular exercise routine, which included exercising for about 3 minutes during the break time provided by the company every day to prevent stiff shoulders and back pain. We also allowed the control group to use the AI-assisted health program after the 12-week intervention.

**Figure 1:**
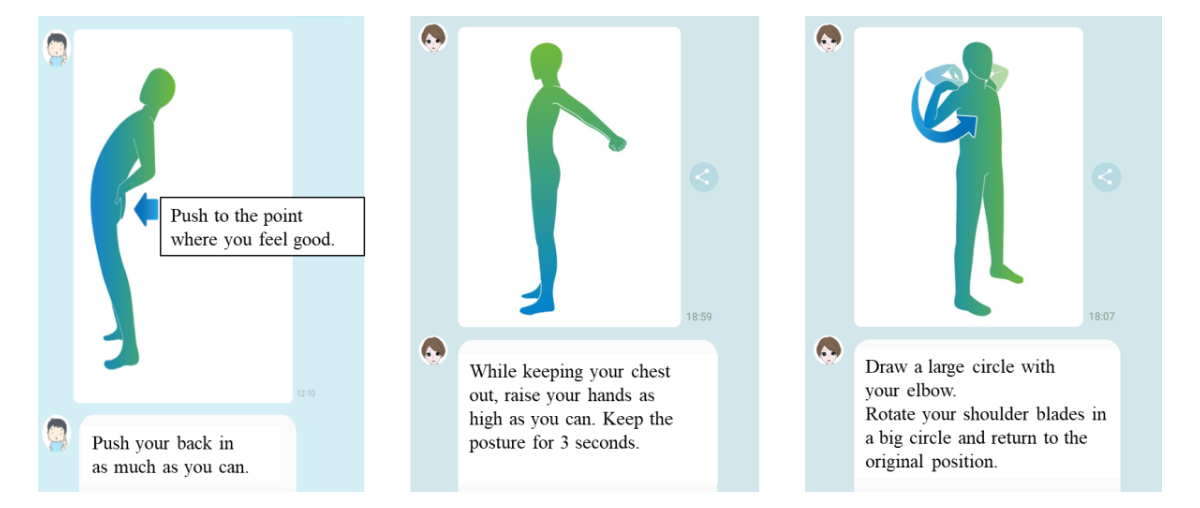
Examples of exercises with instructions from the artificial intelligence–assisted health program.

The AI-assisted chatbot was programmed to send the users messages with the exercise instructions and some tips on what they can do in their daily lives to improve those symptoms. The messages were sent every day at a fixed time through LINE. The notification time could be changed by the users to a time convenient for them. The participants could finish their exercise within 1 minute each day. The program is interactive and the participants can respond to the messages by using a simple selection list; the chatbot offers them tailored replies depending on their responses. The exercise provided by the program had 3 components: stretching [45-47], maintaining good posture [48,49], and mindfulness [21] (Figure 1). When the participants interrupted the exercise, the chatbot motivated them to continue exercising. The program we used in this study was named secaide Ver.0.9, which was patented and has been created and developed since June 2017 by Travoss Co, Ltd and an orthopedist who is specialized in musculoskeletal disorders. Until we performed this interventional study, this program had not been used or evaluated previously. During the intervention period, we notified participants that occupational health staff at the company would respond to inquiries about changes in their physical conditions and the company that provided the program would reply to technical questions about accessing the program, but we did not provide any human support such as specific advice on how to perform the exercises or recommendation to continue the exercise.

### Adherence to the Program

We counted the number of participants who continued to access and reply the chatbot’s messages at least once every 3 days and excluded the participants who did not access and reply for 3 weeks in a row. Then, we calculated the adherence rate by dividing the abovementioned count by the number of participants at the start of the intervention.

### Outcomes

In this study, we set 2 types of outcomes. The first was a subjective assessment of the degree of pain on a scale of 1 to 5; this included subjective ratings of the neck/shoulder stiffness/pain and low back pain at baseline and after 12 weeks. A score of 4 or more was defined as severe pain. The second was a subjective assessment of whether there was an improvement. The participants were asked to subjectively rate whether their pain had improved after 12 weeks; they chose from the following options: improved, slightly improved, unchanged, slightly worse, and worse. Those who responded that their pain had improved or slightly improved were defined as the group that showed subjective improvement. All participants answered the questions through a web-based form.

### Statistical Analysis

A linear regression analysis was used to compare the intervention and control groups for change in the subjective pain scores after the program. The odds ratios (ORs) of the intervention group to achieve a subjective pain score of less than 3 compared with those of the control group was estimated using the logistic regression model. In addition, the OR of the subjective improvement of the symptoms compared to the intervention and control groups was estimated using the logistic regression model. All analyses were performed using Stata software (version 16, StataCorp LLC).

## Results

### Study Population

**Figure 2:**
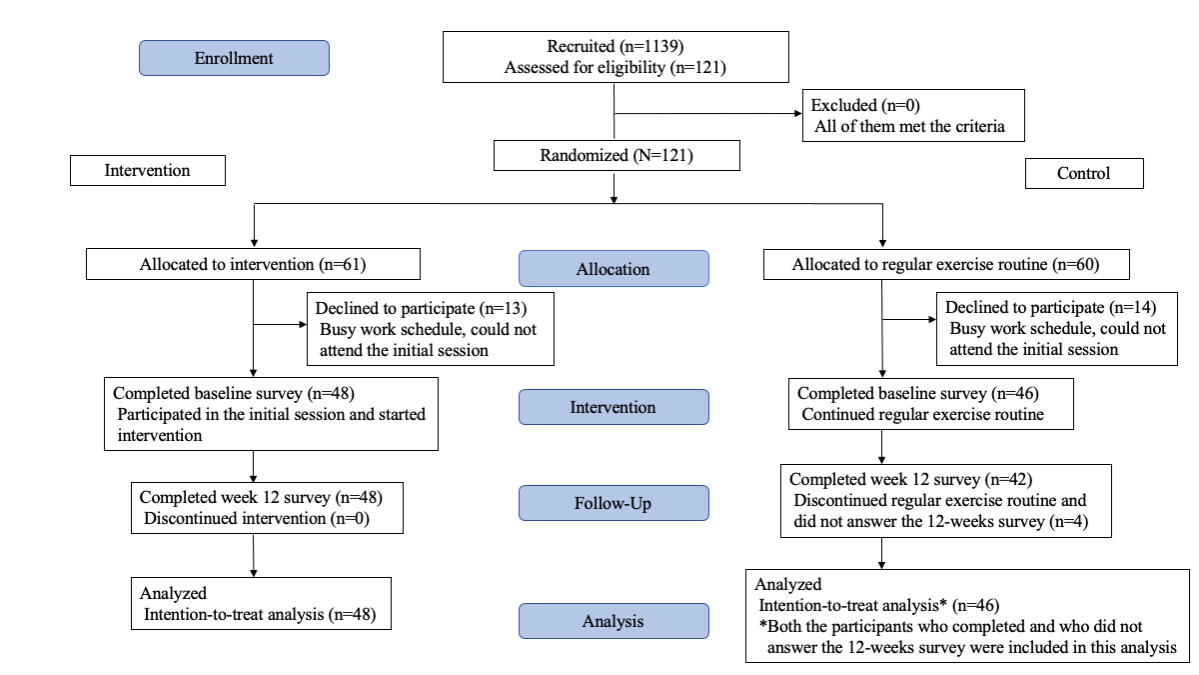
CONSORT (Consolidated Standards of Reporting Trials) flow diagram.

Figure 2 shows the CONSORT flow diagram. A total of 121 employees applied for this study. All the employees were engineers who were engaged in developing or designing precision devices or clerical workers, which meant that they spent most of their working hours doing sedentary work. After we confirmed the eligibility of 121 applicants who wished to participate in the study, we randomly assigned them to an intervention group (n=61) and control group (n=60), because we had to notify the schedule of the initial session during their working hours to the participants. Unfortunately, 13 and 14 applicants allotted in the intervention group and control group, respectively, could not participate in the session or answer the baseline survey. Therefore, the intervention started with 48 and 46 participants in the intervention and control groups, respectively. We could follow up 48 and 42 participants in the intervention and control groups, respectively. Table 1 shows the baseline characteristics of the participants who answered the survey at baseline.

**Table 1:** Baseline characteristics of the participants.

Characteristics			Intervention group (n=48)		Control group (n=46)
Age (years), mean (SD)			41.8 (8.7)		42.4 (8.0)
**Sex, n (%)**					
	Women	9 (19)		13 (28)	
	Men	39 (81)		33 (72)	
Severe neck/shoulder stiffness/pain,^a^ n (%)			34 (71)		31 (67)
Severe low back pain,^a^ n (%)			11 (23)		18 (39)
**Pain level of neck/shoulder stiffness or low back pain, n (%)**					
	3^b^	10 (21)		9 (20)	
	4^b^	24 (50)		19 (41)	
	5^b^	13 (27)		16 (35)	

^a^A score of 4 or more indicates severe pain.

^b^Subjective ratings of the degree of pain on a scale of 1 to 5.

### Adherence to the Program

Of the 48 participants in the intervention group, 47 started the AI-assisted health program and 44 continued the exercise for the entire intervention period. The adherence rate was 92% (44/48) in this study.

### Outcomes

Table 2 shows the results of the outcomes. At 12 weeks, the average pain level of the neck/shoulder stiffness/pain or low back pain was 3.0 (SD 1.1) in the intervention group and 4.0 (SD 0.8) in the control group; the difference was statistically significant (*P*<.001). Each pain level (scores 1 to 5) also showed a statistically significant difference (*P*<.001) between the intervention and control groups. We analyzed the outcomes by dividing them into 2 categories depending on whether symptoms were severe (a score of 4 or more) or not (a score of 3 or less) at 12 weeks. There was a significant difference between the 2 groups (*P*<.001). In the intervention group, the proportion of participants who had severe symptoms decreased from 77% (37/48) at baseline to 33% (16/48) after the intervention. Conversely, in the control group, the proportion of participants who had severe symptoms decreased from 76% (33/46) to 67% (31/46). Furthermore, regarding the subjective assessment of whether there was an improvement, 36 (75%) out of 48 participants in the intervention group and 3 (7%) out of 46 participants in the control group showed improvements (improved, slightly improved), and there was a statistically significant difference (*P*<.001) between the 2 groups.

**Table 2:** Scores of the outcomes after the intervention.

Outcome			Intervention group (n=48)		Control group (n=46)		*P* value
Pain level of the neck/shoulder pain/stiffness or low back pain after the intervention (12 weeks), mean (SD)			3.0 (1.1)		4.0 (0.8)		<.001
**Pain level of the neck/shoulder stiffness/pain and low back pain after the intervention (12 weeks), n (%)**							<.001
	1^a^	2 (4)		0 (0)			
	2^a^	19 (40)		0 (0)			
	3^a^	11 (23)		15 (33)			
	4^a^	10 (21)		16 (35)			
	5^a^	6 (13)		15 (33)			
Presence of severe pain, according to subjective pain scores, Yes (pain score,^a^ 4-5), n (%)			16 (33)		31 (67)		<.001
Achievement of subjective symptom improvement, Yes (improved, slightly improved), n (%)			36 (75)		3 (7)		<.001

^a^Subjective rating of the degree of pain on a scale of 1 to 5.

We performed logistic regression analyses to evaluate the differences in symptoms (neck/shoulder, low back) between the baseline and at 12 weeks after adjusting for age and sex. The difference in the worst pain scores of neck/shoulder pain/stiffness and low back pain between baseline and 12 weeks was –1.12 (95% CI –1.53 to –0.70; *P*<.001) (Table 3). We also examined the OR of the outcomes (Table 3). The participants in the intervention group showed significant improvements in the severity of the neck/shoulder pain/stiffness and low back pain compared to those in the control group (OR 6.36, 95% CI 2.57-15.73; *P*<.001). The OR of the subjective improvement in symptoms at 12 weeks was 43.00 (95% CI 11.25-164.28; *P*<.001).

**Table 3:** The outcomes after the intervention.

Outcome, group		Odds ratio (95% CI)	*P* value
**Difference in the worst pain scores^a^ between baseline and 12 weeks**			<.001
	Control	Reference	
	Intervention	–1.12^b^ (–1.53 to –0.70)	
**Absence of severe^c^ pain according to subjective pain scores**			<.001
	Control	Reference	
	Intervention	6.36^d^ (2.57 to 15.73)	
**Achievement of subjective symptom improvement**			<.001
	Control	Reference	
	Intervention	43.00^d^ (11.25 to 164.28)	

^a^Subjective rating of the degree of pain on a scale of 1 to 5.

^b^The estimate represents a regression coefficient.

^c^A score of 4 or more indicates severe pain.

^d^The estimate represents an odds ratio from a logistic regression model.

## Discussion

### Overview

This RCT showed that the 12-week use of an AI-assisted health program that provides 1 short exercise routine per day significantly improved (1) the subjective symptoms of both neck and shoulder pain/stiffness and low back pain after 12 weeks compared to those at baseline and (2) the subjective assessment of improvement following the 12-week intervention in the intervention group compared to that in the control group. In this study, the intervention group showed a high adherence of 92% (44/48) for the whole intervention period as we hypothesized that this digital intervention would support participants to continue exercising and enhance their adherence to the exercises, resulting in greater improvement in symptoms. High adherence is associated with improvement of the symptoms [22,26,27]. The results seemed to be largely attributed to the high adherence of the participants. Previous reports have shown that adherence to home-based exercise was at most 50%-70% [24,26].

This program was designed to improve exercise adherence. This program has been mainly implemented with both instructions and reminder functions. The chatbot sent a message with the exercise instructions and a corresponding illustration at a fixed time every single day, which also functioned as a reminder feature that sent instructions at a fixed time each day. Some studies have shown that social support is essential for enhancing adherence to home-based exercise [26,50]. Concretely, feedback from experts to patients, interactions between patients and caregivers, and supervision from experts improve adherence [24,51]. Digital interventions were more effective with human support [52]. In addition, further support such as phone calls, email reminders, and text messages was used to promote engagement in digital interventions [28-33]. Email reminders increased exercise adherence [53,54]. Interaction through the chatbot app of this program may have also given participants a sense of support. Although the participants were not supervised or provided feedback by the experts, the service provided both good and bad examples of how the exercise was supposed to be performed. Besides, they were motivated from time to time to continue with the exercise every day. Therefore, the participants might have felt supervised. In that respect, the program also had a monitoring function. The positive influence of these functions was consistent with the fact that the roles of human caregivers were essential for patients to continue with prescribed exercises. The beneficial features of this program were that we used a chat app, LINE, and the daily exercise time was the shortest used to date.

LINE is the most commonly used chat app in Japan and is similar to Facebook messenger, WhatsApp in the United States and Europe, and WeChat in China. This app allows users to send messages with emoji and stickers to individuals or groups and make voice or video calls. It is used mainly in Japan, Taiwan, and parts of Southeast Asia, which has approximately 86 million users (2020) [55]. Many users look at the app once they receive a message automatically. Therefore, when the participants received a message from the chatbot, they checked the messages immediately, which led them to continue the program. The high rate of adherence to the program in this intervention may also be attributed to the simple and extremely short exercise offered in this program compared to that offered in previous studies. In this study, daily exercises could be performed within 1 minute per day, and their effects were evident within a short time. Exercising for an average duration of 10 minutes per day significantly improved low back pain [15]. Exercises performed for 10-15 minutes each day 3-5 times a week were effective for stiff shoulders [16,18]. The duration of use of a mobile app to individually manage chronic neck and back pain was recommended for 20-30 minutes per day in another study [56].

We performed this intervention for only 12 weeks, which is considered a reasonable period of time in terms of the effects on the musculoskeletal symptoms, adherence to the program, and follow-up of the participants. Long-term improvement in musculoskeletal symptoms can be expected with continued use of the program, but the adherence to the program could decline in the population. If the exercise is used only during the intervention period, the symptoms may gradually worsen again unless the patients continue the same exercises by themselves even after the end of the interventional period. According to several systematic reviews [22,28,34], the duration of the intervention ranges from 3 weeks to 12 months, but the relationship between the duration of the intervention and the outcome is not clear [28]. Although a few studies have mentioned the long-term effects of exercises for musculoskeletal symptoms, the exercises for low back pain combined with the program aimed at keeping motivation showed significant improvements in pain and function at 5-year follow-ups and about 60% adherence for both the intervention and the control groups [57].

The target population for this study was workers at the same company. They were usually engaged in jobs that required the use of computers and were accustomed to using smartphones on a daily basis. In other words, they had high computer/internet literacy. The program using LINE was highly compatible and it was easy to continue for them. Since this intervention was conducted as a part of the company’s health promotion activities, they were dedicated to adhere to and continue the program even though there was no requirement to do so. Hence, it is understandable that the results may differ depending on the intervention population such as community residents, older adults, and outpatients.

A secondary effect of introducing mobile health and eHealth into health promotion activities is that people can easily access and utilize evidence-based health information for their own health care and, in other words, they can improve their own health literacy [58]. In the case of workers, mobile health can be used as an opportunity for those who have difficulty paying attention to their own health management owing to their busy schedules or other reasons. We are not sure which elements of this intervention, including the exercises themselves, were clearly effective. Although we used a specific program in this study, apps or internet services with similar functions such as sending easy and simple instructions every day at a fixed time may be effective in relieving musculoskeletal symptoms.

### Limitations

There are several limitations in this study. First, no information was collected on the possible causes and diagnosis of neck/shoulder pain/stiffness and low back pain. The status of treatment such as the use of analgesics was also unknown. The improvement in pain observed in the intervention group may be due not only to the intervention but also to the changes in the treatment and occupational factors. However, since this study is an RCT, we believe that such effects are not necessarily crucial. Second, 13 participants in the intervention group and 14 in the control group dropped out after being randomly allotted before the start of the program. Hence, we could not even obtain their baseline data. Since we performed this intervention as a part of the health promotion activities at workplace during working hours, we needed to provide them the schedule for the introduction of the briefing session of the study in advance to have them participate in it. This matter forced us to randomize the participants as soon as they had enrolled as participants and had checked their eligibility to participate in this study, which resulted in dropouts prior to participation in the intervention. Further, some individuals could not participate the program because it did not align with their schedules; such withdrawal of participation was inevitable. Third, the definition of adherence differs in literature and is often not clearly defined. Although some studies have relied on participants’ self-reporting [26], we could estimate the adherence by the access history of the program, as this study was a digital intervention. However, we could only know if they had accessed the program but we could not know if they had done the exercises. It is important to note that the adherence rate is different from the rate of exercising. However, we believe that many of the participants were able to do the exercises because the results showed improvement in the musculoskeletal symptoms. Fourth, this study was unblinded because of the characteristics of the intervention. The influence of the Hawthorne effect should be considered. Although we also provided the AI-assisted health program to the control group after completing the intervention for the intervention group and we did not reveal to the intervention group that they were the intervention group, it cannot be denied that the population of the intervention group could infer that they had been selected and should be able to improve because they were doing the exercises every day. Finally, regarding the outcome, we only adopted the subjective symptoms of neck/shoulder pain/stiffness and low back pain. The subjective symptoms are highly variable depending on their condition. We recruited the target papulation as those who had the symptoms above a certain level in the health survey in the past, but some participants had weak subjective symptoms in the baseline survey. We did not exclude such participants in this study.

### Conclusions

This RCT showed that an intervention with simple and short exercises provided by an AI-assisted health program via participants’ mobile phones’ text messaging app for 12 weeks improved both neck and shoulder stiffness and low back pain. Digital health programs could help busy workers continue with their exercise routines easily without the need for frequent and direct contact with medical professionals. Further studies are needed to identify the elements of the AI-assisted health program that worked.

## Appendix

Multimedia Appendix 1 CONSORT-eHEALTH checklist (V 1.6.1).

## Abbreviations

AI: artificial intelligence
CONSORT: Consolidated Standards of Reporting Trials
OR: odds ratio
RCT: randomized controlled trial
